# Inflammatory pathways confer resistance to chemoradiotherapy in anal squamous cell carcinoma

**DOI:** 10.1038/s41698-024-00585-y

**Published:** 2024-04-23

**Authors:** D. Martin, F. Rödel, S. Hehlgans, M. Looso, P. K. Ziegler, M. Fleischmann, M. Diefenhardt, L. Fries, G. Kalinauskaite, I. Tinhofer, D. Zips, C. Gani, C. Rödel, E. Fokas

**Affiliations:** 1 Department of Radiotherapy and Oncology, Goethe University Frankfurt, University Hospital, Frankfurt, Germany; 2 German Cancer Consortium (DKTK), Partner Site Frankfurt, A Partnership between DKFZ and University Hospital Frankfurt, Frankfurt, Germany; 3 Frankfurt Cancer Institute (FCI), Goethe University Frankfurt, Frankfurt, Germany; 4 Max Planck Institute for Heart and Lung Research, Bioinformatics Core Unit, Bad Nauheim, Germany; 5 Dr. Senckenberg Institute of Pathology, Goethe University Frankfurt, University Hospital, Frankfurt, Germany; 6 Department of Radiooncology and Radiotherapy, Charité University Hospital Berlin, Berlin, Germany; 7 German Cancer Consortium (DKTK), Partner Site Berlin, A Partnership between DKFZ and Charité University Hospital Berlin, Berlin, Germany; 8 Eberhard Karls University, Tübingen, University Hospital Tübingen, Department of Radiation Oncology, Tübingen, Germany; 9 German Cancer Consortium (DKTK), Partner Site Tübingen, A Partnership between DKFZ and University Hospital Tübingen, Tübingen, Germany; 10 Department of Radiation Oncology, Cyberknife and Radiotherapy, Center for Integrated Oncology Aachen Bonn Cologne Duesseldorf (CIO ABCD), University of Cologne, Faculty of Medicine and University Hospital of Cologne, Cologne, Germany

**Keywords:** Anal cancer, Tumour biomarkers

## Abstract

Anal squamous cell carcinoma (ASCC) is associated with immunosuppression and infection with human papillomavirus (HPV). Response to standard chemoradiotherapy (CRT) varies considerably. A comprehensive molecular characterization of CRT resistance is lacking, and little is known about the interplay between tumor immune contexture, host immunity, and immunosuppressive and/or immune activating effects of CRT. Patients with localized ASCC, treated with CRT at three different sites of the German Cancer Consortium (DKTK) were included. Patient cohorts for molecular analysis included baseline formalin fixed paraffin embedded biopsies for immunohistochemistry (*n* = 130), baseline RNA sequencing (*n* = 98), peripheral blood immune profiling (*n* = 47), and serum cytokine measurement (*n* = 35). Gene set enrichment analysis showed that pathways for IFNγ, IFNα, inflammatory response, TNFα signaling via NF-κB, and EMT were significantly enriched in poor responders (all *p* < 0.001). Expression of interferon-induced transmembrane protein 1 (IFITM1), both on mRNA and protein levels, was associated with reduced Freedom from locoregional failure (FFLF, *p* = 0.037) and freedom from distant metastasis (FFDM, *p* = 0.014). An increase of PD-L1 expression on CD4+ T-cells (*p* < 0.001) and an increase in HLA-DR expression on T-cells (*p* < 0.001) was observed in the peripheral blood after CRT. Elevated levels of regulatory T-cells and CXCL2 were associated with reduced FFLF (*p* = 0.0044 and *p* = 0.004, respectively). Inflammatory pathways in tissue in line with elevated levels of regulatory T-cells and CXCL2 in peripheral blood are associated with resistance to CRT. To counteract this resistance mechanism, the RADIANCE randomized phase-2 trial currently tests the addition of the immune checkpoint inhibitor durvalumab to standard CRT in locally advanced ASCC.

## Introduction

Anal squamous cell carcinoma (ASCC) is a rare disease with an annual incidence of 2–3 per 100,000 inhabitants^1^. In recent years, an increase in the incidence of ASCC has been reported^2^. The main risk factors for ASCC are immunosuppression and infection with human papillomavirus (HPV), which can be found in up to 95% of patients^3^. Following HPV viral DNA integration into host cells, oncoproteins E6 and E7 induce inactivation of tumor suppressors such as retinoblastoma protein and p53. Moreover, activating mutations of *PIK3CA* and mutations of genes important for DNA damage repair (*ATM, BRCA 1/2, HUWE1*), chromatin remodeling (*EP300, SMARCB1*), and the Wnt/β-catenin signaling (*FAM123B9*) have been frequently reported in ASCC, whereas clinically relevant alterations for *KRAS*, *NRAS*, *BRAF*, *EGFR* are rare^4–7^.

Standard treatment for localized ASCC is primary chemoradiotherapy (CRT). Radical surgery is reserved as salvage for locoregional failure. Several large randomized trials have tested various forms of induction or consolidation chemotherapy in addition to concurrent CRT, but failed to show oncological benefits. Thus, the use of 5-fluorouracil (5-FU) plus mitomycin c (MMC) CRT remains the standard of care^8,9^.

Smaller tumors without lymph node involvement (cT1-2N0M0) generally have a favorable prognosis after standard CRT, but incomplete responses or recurrences occur in up to 40% of patients with locally-advanced ASCC (cT3-4 and/or cN+)^10,11^. Well established clinical prognostic factors include primary tumor size, lymph node involvement, and sex^11–13^. In addition, expression of p16^INK4A^ (surrogate for HPV) and CD8+ tumor infiltrating lymphocytes (TILs) have recently been established as molecular prognostic markers^14,15^. Given the relatively rare incidence of ASCC, a comprehensive molecular characterization of ASCC is lacking, and genomic profiling as part of The Cancer Genome Atlas (TCGA) project has not been reported. Here, we present results from multidimensional analyses of ASCC to gain a better molecular understanding of response to CRT, to develop prognostic/predictive markers for individual treatment efficacy and to help guide future treatment strategies based on subgroups identified.

## Results

### Patient characteristics and oncological outcome

Baseline demographics and clinical characteristics of the full cohort of 130 patients are summarized in Table 1; 70 (54%) patients had cT1-2N0M0 disease, and 60 (46%) patients were diagnosed with locoregionally advanced disease (cT3N0M0 or cTanyN+M0). A total of 22 patients (17%) were HIV-positive; these were mainly male (19/22), and significantly younger than HIV-negative patients (median 52 vs. 59 years; *p* < 0.001).

**Table 1:** Patient’s characteristics

		Median (range) or *n* (%)
Age, years		58 (26–86)
Sex	Male	56 (43)
	Female	74 (57)
HIV-Status	Positive	22 (17)
	Negative	100 (77)
	Unknown	8 (6)
T-Stage	T1	31 (24)
	T2	55 (42)
	T3	34 (25)
	T4	14 (10)
N-Stage	N0	84 (65)
	N+	46 (35)
Grading	G1	6 (5)
	G2	86 (66)
	G3	36 (28)
	unknown	2 (1)
*Radiotherapy*		
RT modality	3D	45 (35)
	IMRT	85 (65)
Total dose (Gy)		59.4 (45–65)

*HIV* human immunodeficiency virus, *Gy* Gray, *RT* radiotherapy, *3D* 3-dimensional radiotherapy, *IMRT* intensity modulated radiotherapy.

With a median follow-up of 48.5 (IQR, 24.5–70.25) months, cCR was achieved in 115/130 (88%) patients, a total of 27 (21%) patients either had residual tumor at first re-staging or developed a locoregional recurrence after initial cCR, 12 patients (9%) developed distant metastasis during follow-up; 5-year FFLF was 76.5% (95% CI, 69–85%), and 5-year FFDM was 89.2% (95% CI, 83–96%).

### Interferon-associated pathways are enhanced in poor responders

**Fig. 1:**
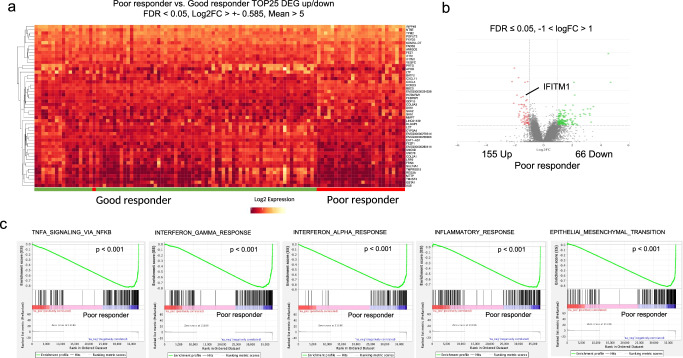
Results of RNA seq analysis and gene set enrichment analysis. **a** Top 25 differentially expressed genes between poor responders and good responders. **b** Volcano plot of the RNA Seq data dichotomized according to poor response (red) and good response (green). **c** Enrichment plots from the GSEA tool showing that in poor responders the pathways TNFα Signaling via NF-κB, Interferon gamma and alpha response, inflammatory response, and epithelial mesenchymal transition (EMT) are significantly enriched.

After rigorous quality control of the RNA sequencing data in 130 baseline tumor samples, 98/130 (75%) were considered as suitable and selected for further analysis (Fig. 1a, GSEA254331). Patient characteristics and treatment outcomes of this group are summarized in Supplementary Table 1. Gene set enrichment analysis showed that pathways for interferon gamma and alpha response (IFNγ and IFNα, *p* < 0.001, respectively), inflammatory response (*p* < 0.001), epithelial mesenchymal transition (EMT, *p* < 0.001), and tumor necrosis factor alpha (TNFα) signaling via NF-κB (*p* < 0.001), were significantly enriched in poor responders to CRT (Fig. 1c). Also, DEG analysis revealed that interferon induced transmembrane protein 1 (IFITM1) was significantly upregulated in poor responders (log2FC −1.42; *p*adj 0.005, Fig. 1b), and also part of the enriched hallmark gene sets IFNα response and inflammatory response (Supplementary Table 3).

### IFITM1 expression is associated with poor prognosis

**Fig. 2:**
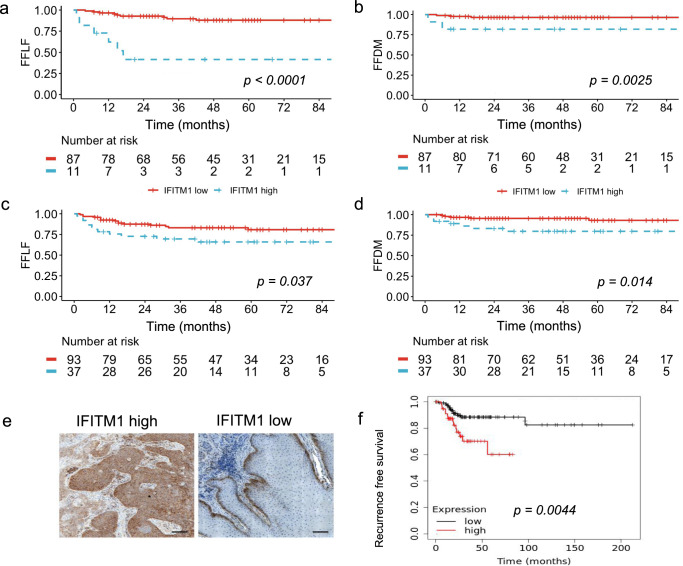
IFITM1 expression is associated with worse outcome. After identification of an ideal cut-off using maximized rank statistics, a high expression of interferon induced transmembrane protein 1 (IFITM1) in RNA seq was associated with significantly reduced freedom from local failure (FFLF) (**a**), and freedom from distant metastasis (FFDM) (**b**). Using immunohistochemistry for IFITM1, a high semiquantitative weighted score for IFITM1 was associated with reduced FFLF (**c**) and FFDM (**d**). **e** Example stainings for IFITM1 high and low tumors. **f** Recurrence free survival in the The Cancer Genome Atlas (TCGA) cervical cancer cohort according to IFITM1 expression. The plot was generated using https://www.kmplot.com.

Using the R survminer package to create an optimal cut-off, a high expression of IFITM1 was associated with significantly reduced FFLF (*p* < 0.0001, Fig. 2a) and FFDM (*p* = 0.025, Fig. 2b) in the RNAseq cohort. To validate this finding at the protein level, we stained pretreatment tumor samples of the whole cohort (*n* = 130) for IFITM1 (Fig. 2e). Median IFITM1 weighted score was 6. The cohort was divided accordingly into a high score (score >6) and a low score (≤6); 37 (28%) patients had a high expression, and 93 (72%) a low expression. IFITM1 was not associated with T-stage, N-stage, sex, and HIV-status (Table 2). A high immunohistochemical expression of IFITM1 was associated with reduced FFLF and FFDM (*p* = 0.037, *p* = 0.014, respectively, Fig. 2c, d).

**Table 2:** Association of IFITM1 expression with clinicopathologic parameters

	IFITM1 low (%)	IFITM1 high (%)	*p* Value
Sex			
Male	36 (39)	20 (54)	
Female	57 (61)	17 (46)	0.11
HIV			
Negative	75 (81)	25 (67)	
Positive	14 (15)	8 (22)	
Unknown	4 (4)	4 (11)	0.21
T Stage			
T1	23 (24)	8 (22)	
T2	39 (42)	16 (43)	
T3	21 (23)	11 (30)	
T4	10 (11)	2 (5)	0.69
N Stage			
N0	60 (65)	24 (65)	
N+	33 (35)	13 (35)	0.9
p16^INK4A^			
Low	41 (44)	14 (38)	
High	52 (56)	23 (62)	0.5
CD8			
Low	73 (78.5)	28 (75.7)	
High	20 (21.5)	9 (24.3)	0.7
TPS			
0	47 (81)	11 (31)	
≥1	11 (19)	24 (69)	0.04
CPS			
<10	72 (82)	22 (63)	
≥10	16 (18)	13 (37)	0.03

*HIV* human immunodeficiency virus, *TPS* tumor proportion score, *CPS* combined positive score.

Next, we assessed IFITM1 using the TCGA cervical cancer dataset as an example for another HPV-driven squamous cell carcinoma. In line to the findings in our ASCC RNAseq cohort, we found a significant association between high expression of IFITM1 and reduced recurrence-free survival in the TCGA cervical cancer dataset (Fig. 2f).

Considering the potential relevance of IFITM1 for immunoregulation, we assessed whether it correlates with key markers of the ASCC immune contexture, such as p16^INK4A^, CD8 + TIL, and PD-L1 status. The median p16^INK4A^ score was 6, and patients were divided in the same manner as for IFITM1; 75 (58%) patients had high expression, and 55 (42%) low expression of p16^INK4A^. Also, 29 (22%) of patients had high, and 101 (78%) had low CD8 + TIL infiltration. A high expression of IFITM1 was associated with a PD-L1 CPS ≥ 10 (*p* = 0.03), and a TPS ≥ 1 (*p* = 0.04) (Table 2), whereas no significant association between IFITM1 and expression of p16^INK4A^ or CD8 + TIL was noted. In accordance to our previous reports, we confirmed that high p16^INK4A^ and high CD8 + TIL expression were associated with improved FFLF (*p* = 0.006 and *p* = 0.042, respectively, Supplementary Fig. 3), while FFDM was not significantly impacted. After creating a combined variable with p16^INK4A^ and CD8 we found that patients with a high expression in both markers had an excellent prognosis with no events of locoregional failure, while patients with a low expression of both parameters had very poor local control (*p* = 0.019, Supplementary Fig. 4A). We also analyzed combinations of IFITM1 with p16^INK4A^ or CD8. The few patients with a low p16^INK4A^ score and a high IFITM1 expression had a significantly decreased FFLF (*p* < 0.001, Supplementary Fig. 4B), whereas the combination of high IFITM1 expression and low CD8 TIL was associated with a significantly decreased FFLF (*p* = 0.0018, Supplementary Fig. 4C). Additionally, there was no significant association between p16^INK4A^ and expression levels of IFITM1 in the RNA seq cohort (*p* = 0.68). Due to possible influences on the immune contexture, we also investigated if HIV-status affects any of the analyzed markers. We found no significant association between HIV-status and either p16^INK4A^ (*p* = 0.5), CD8 (*p* = 0.35), IFITM1 (*p* = 0.41), TPS (*p* = 0.57) or CPS (*p* = 0.7). Additionally, there was no significant association between HIV-status und IFITM1 expression in the RNAseq cohort (*p* = 0.59). We also conducted multivariate cox regression analysis for FFLF and FFDM including established clinical parameters like T-stage and N-stage and included p16^INK4A^ and IFITM1. A high IFITM1 score was associated with a reduced FFLF (HR 2.79; *p* = 0.009) and FFDM (HR 4.39; *p* = 0.012) independent of T-stage, N-stage and p16^INK4A^ (Table 3).

**Table 3:** Multivariate Cox regression analysis for freedom from locoregional failure (FFLF) and freedom from distant metastasis (FFDM) using T stage, N-stage and IFITM1 and p16^INK4A^ scores assessed via immunohistochemistry

	Univariate analysis	Multivariate analysis		
	*p*	HR	95% CI	*p*
FFLF				
T3/4 vs. T1/T2	<0.001	2.49	1.047–6.186	0.048
N+ vs. N0	<0.001	3.17	1.232–8.159	0.017
IFITM1 high vs. low	0.04	2.79	1.287–6.040	0.009
p16^INK4A^ high vs. low	0.01	0.43	0.189–0.985	0.045
FFDM				
T3/4 vs. T1/T2	0.005	3.41	0.79–14.59	0.099
N+ vs. N0	0.005	3.98	0.92–17.14	0.064
IFITM1 high vs. low	0.023	4.39	1.38–13.95	0.012
p16^INK4A^ high vs. low	0.08	–	–	–

To assess the role of EMT, we stained pretreatment samples for E-Cadherin and Vimentin (Supplementary Fig. 6). Scores were dichotomized in high (weighted score >6) and low (WS ≤ 6). We found a significantly worse OS in patients with a low expression of E-Cadherin (*p* = 0.017), while there was only a non-significant trend towards a worse FFLF (*p* = 0.09) and FFDM (*p* = 0.052). For Vimentin, a significantly worse FFLF (*p* = 0.014), FFDM (*p* = 0.028) and OS (*p* = 0.035) was noted for patients with a high intratumoral expression of vimentin (Supplementary Fig. 6). A low expression of E-Cadherin and a high expression of Vimentin was furthermore associated with advanced (cT3-4) tumors (*p* = 0.004, *p* = 0.013), while only a high expression of E-Cadherin was associated with cN+ tumors (*p* = 0.008). Additionally, there was no association of E-Cadherin or Vimentin with IFITM1, p16^INK4A^, TPS or CD8.

### Regulatory T cells in the peripheral blood are associated with poor prognosis

**Fig. 3:**
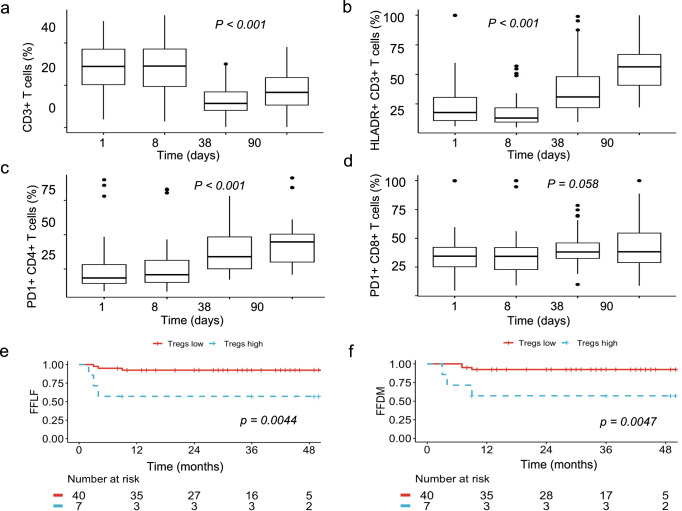
Changes in the peripheral blood during chemoradiotherapy. From start to the end of CRT, and first follow-up, the amount of CD3+ T cells in the peripheral blood was significantly reduced (**a**), while the expression of human leukocyte antigen—DR isotype (HLADR) on CD3+ T-cells increased significantly (**b**). PD1 expression on CD4+ T-cells was significantly higher at the end of CRT (**c**), while PD1 expression on CD8+ T-cells remained stable (**d**). A high proportion of CD4+ T cells that express CD25 and FoxP3 (Tregs—regulatory T cells) is associated with significantly decreased freedom from local failure (FFLF) and freedom from distant metastasis (FFDM) (**e**, **f**).

To assess the peripheral blood immune profile, we conducted FACS analysis in 47 consecutive ASCC patients with available peripheral blood samples (Supplementary Tables 4–7). The proportion of CD3+HLA-DR+ T-cells was significantly higher in male than in female patients at baseline (median 25.3% vs. 13.8%, *p* = 0.0021). Additionally, in HIV positive patients, CD3+HLA-DR+ T-cells were also significantly elevated at baseline (median 40.4% vs. 13.9%, *p* < 0.001), whereas no differences in the expression of PD1 on CD4+ or CD8+ T-cells was noted (*p* = 0.08 and *p* = 0.8, respectively). There were no other significant associations with T-stage, N-stage, or sex. The longitudinal measurements showed a significant reduction of CD3+ T-cells during CRT (*p* < 0.001, Fig. 3a), while the proportion of CD3+ T-cells expressing HLA-DR increased significantly from start of treatment until the first follow-up visit (*p* < 0.001, Fig. 3b). Additionally, the expression of PD1 on CD4+ T-cells increased significantly during CRT, while the PD1 expression on CD8+ T-cells showed no significant changes (Fig. 3c, d). These changes during treatment were significant regardless of the HIV- status (Supplementary Fig. 5). In this subset of 47 patients, 6 cases of locoregional failure, and 6 cases of distant metastasis were diagnosed during follow-up. Using the proportion of CD4+ T-cells that express CD25 and FoxP3 (regulatory T cells; Tregs) as a continuous variable, a cox regression showed a significantly reduced FFLF and FFDM for patients with a higher proportion at baseline (LF: HR 1.44; 95% CI: 1.05–1.97; *p* = 0.024; DM: HR 1.63; 95% CI: 1.15–2.31; *p* = 0.007). Using maximally selected rank statistics the ideal cut-off for Tregs was calculated at 5.2% (of CD3+ T-cells). After dichotomization, 7 (15%) patients were deemed to have high Tregs at baseline, and this was associated with a reduced FFLF (*p* = 0.0044, Fig. 3e) and FFDM (*p* = 0.0047, Fig. 3f). There were no significant differences between the levels of Tregs and HIV-status (*p* = 0.49).

### CXCL2 levels in the peripheral blood are associated with neutrophilia and poor prognosis

**Fig. 4:**
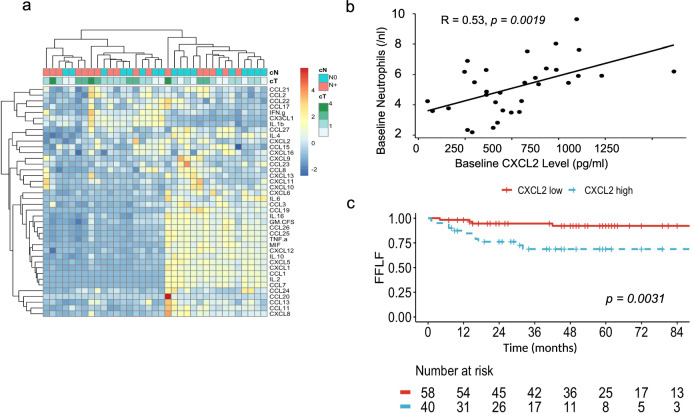
CXCL2 in the peripheral blood and in the RNA seq analysis and outcome. **a** Heatmap showing the concentration of several cytokines for 35 patients before initiation of treatment. On the top, N-stage and T-stage are annotated. **b** Serum CXCL2 levels at baseline are significantly correlated with neutrophil levels. **c** Kaplan–Meier plots that show that an increased expression of CXCL2 in the RNAseq dataset (*n* = 98) is associated with impaired freedom from local failure (FFLF).

We conducted baseline multiplex cytokine/chemokine measurements in 35 consecutive patients for whom peripheral blood serum samples were available (Fig. 4a). A high level of CXCL2 was significantly associated with higher T-stage (*p* = 0.0095) and neutrophil levels in the peripheral blood at baseline (*R* = 0.53, *p* = 0.0019, Fig. 4b). There was no significant correlation between CXCL2 levels and neutrophil to lymphocyte ratio (NLR) at baseline (*R* = 0.32, *p* = 0.075). Due to the small sample size, no significant correlation of CXCL2 levels was noted for FFLF or FFDM, however, combined locoregional and distant failure was significantly associated with CXCL2 levels (HR for an increase of 10 pg/ml 1.02; 95% CI 1.01–1.03, *p* = 0.004). Moreover, high expression of CXCL2 in tumor tissue (RNAseq dataset) was associated with a reduced FFLF (*p* = 0.0031, Fig. 4c).

## Discussion

To the best of our knowledge, we present the largest RNA sequencing cohort in patients treated with standard CRT for localized ASCC for whom clinically annotated data and long-term oncological outcome is available. Gene set enrichment analysis revealed that hallmark pathways associated with chronic inflammation (IFNγ; IFNα, inflammatory response, TNFα signaling via NF-κB) and EMT are significantly enriched in patients with poor response and prognosis. IFN signaling is at the core of immune regulation and generally works in an immune-stimulatory, proapoptotic and antiproliferative fashion to confer antiviral and antitumor properties to the host. However, it is well known that chronic IFN signaling can also lead to treatment resistance in tumors, e.g. via regulation of multigenic resistance programs to immune checkpoint blockade, including upregulation of PD-L1, on cancer cells^16,17^.

We here showed that the interferon-induced transmembrane protein 1 (IFITM1) is an adverse prognostic factor for FFLF and FFDM in ASCC, both on mRNA and protein expression levels, and acts independently of established prognostic factors like T-stage and N-stage. IFITM1 is part of the IFITM family of transmembrane proteins that exert antiviral properties by inhibiting the entry of viruses into cells, but may also play a pivotal role regulating innate antiviral and inflammatory as well as adaptive T-cell and B-cell responses^18,19^. Recent evidence showed that high expression of IFITM1 was associated with therapeutically resistant and aggressive disease courses for lung, esophageal and colon cancer, while a protective role was reported in cervical cancer and gastric cancer^20–23^.

The molecular mechanism of IFITM 1 in cancer generally remains poorly examined, but may include, among others, regulation of matrix metalloproteinases, caveolin-1, and other proteins associated with cell migration and adhesion^24–26^. Interestingly, a recent study in oral squamous cell carcinoma (also HPV-related) found that siRNA knockdown of IFITM1 sensitized tumor cells to ionizing radiation in vitro, indicating that IFITM1 may also be involved in DNA damage repair, although the mechanism remains unclear^27^. Here we demonstrate that high expression of IFITM1 in ASCC was associated with high PD-L1 expression on tumor cells (TPS), and on immune and tumor cells combined (CPS), which is in line with the notion of an immunoregulatory role of IFITM1 in ASCC^18,28^. This may, directly or indirectly, limit tumor immune activation. The lack of association between IFITM1 and p16 was surprising as it has been reported that IFITM1 is downregulated by HPV^29^. It is possible that the occurrence of infection is distinctively different to the state of tumorigenesis. It is also noteworthy that we found no differences in the composition of the tumor immune microenvironment according to HIV status, which is in line with the literature^30^.

Additionally, we further examined the role of EMT via immunostaining for classical EMT markers E-Cadherin and Vimentin. To the best of our knowledge this has not been analyzed in ASCC yet. A high expression of Vimentin was associated with impaired FFLF and FFDM in line with similar data in head and neck squamous cell carcinoma^31,32^.

In general, baseline leukocytosis and neutrophilia in the peripheral blood is a well-established negative prognostic factor in many tumor types. Recently, we could show that peripheral leukocytosis was inversely correlated with intratumoral CD8+ T-cells infiltration and associated with worse outcome after CRT in ASCC^33,34^. No detailed prospective immune monitoring studies have been reported for ASCC patients to date. Therefore, we assessed serial peripheral blood samples at baseline, during, at completion, and at first response assessment 6–8 weeks after CRT by use of multiparametric flow cytometry of PBMC. In line with reports in other tumor types^35–37^, a reduction of CD3+ T-cells and lymphopenia were observed after CRT. Interestingly, the remaining T-cells strongly expressed the activation marker HLA-DR which mediates antigen presentation and was suggested as possible biomarker to predict response to chemotherapy in breast cancer^38,39^. Conversely, CD4+ T-cells showed a marked increase in PD-L1 expression at CRT completion, in line with the observation that fractionated radiotherapy can lead to an upregulation of PD-L1^40,41^. Thus, the observed HLA-DR activation and PD-L1 upregulation on CD4+ T-cells following CRT could be reflections of both immunosuppressive and immune activating effects of fractionated radiotherapy^42^. Whereas there were no differences in the tumor microenvironment between HIV-positive and -negative patients, we found a marked increase of CD3+HLA−DR+ cells at baseline in HIV+ patients. Nevertheless, the changes in the peripheral blood immune profile were again similar regardless of HIV status, supporting the notion that HIV+ patients under antiretroviral therapy can be treated like HIV- patients with CRT. Moreover, a higher proportion of CD25+/FoxP3+CD4+ T-cells (Tregs) at baseline was associated with a significantly reduced FFLF and FFDM in our series, indicating that a systemic immunosuppressive state is associated with poor prognosis in ASCC. Of note, in a previous work we found that a high intratumoral expression of FoxP3+ was associated with improved disease-free survival^14^—suggesting that there are differences in the role of systemic and intratumoral Tregs. Regarding other types of immune cells, the role of tumor associated macrophages in the microenvironment or myeloid derived suppressor cells in the peripheral blood and tumor microenvironment in anal cancer is still unclear. The differences in baseline CD3+ T-cells between male and female patients were most likely due to the fact that the vast majority of HIV+ patients in this cohort were male. Nevertheless, we found no differences in the changes of CD3+ T-cells, CD3+HLA-DR+ T-cells and CD4+PD1+ T-cells according to HIV-status. To gain more insights into the peripheral blood immune profile, several other markers, e.g. immune checkpoint molecules like LAG3, CTLA-4 or TIM-3, should be assessed in further flow cytometry studies of ASCC patients.

Finally, multiplex cytokine/chemokine measurements were conducted in a subset of 35 consecutive patients. CXCL2, a neutrophil chemo-attractant that is involved in chronic inflammation and tumor progression, was identified as negative prognostic factor after CRT in peripheral blood. Moreover, high expression of CXCL2 in tumor tissue in the RNAseq dataset was associated with significantly reduced FFLF. This again sheds light on the link between chronic inflammation in the tumor tissue and changes in the peripheral blood leading to worse treatment outcome in ASCC.

This study has several limitations. First, the retrospective nature of data collection could lead to selection bias. Second, immune phenotyping and cytokine measurements of blood samples were only available in a subset of patients, and cytokine measurement was restricted to baseline analysis to identify predictive markers. Third, the markers Foxp3 and CD25 alone are limited to demonstrate that these cells are Tregs, as a transient upregulation of FoxP3 on T-cells is possible^43^. Fourth, due to the rarity of the disease all patients were treated within a large timeframe, and it is known that PD-L1 staining decreases with sample age^44^. Fifth, p16^INK4A^ is only a surrogate for HPV infection but could also be upregulated due to other causes. Nevertheless, it is already known that infection with HPV16 either as a mono infection or dual/triple infection is the main driver of p16^INK4A^ expression in ASCC accounting for more than 90% of cases^45^.

Despite the novelty of the findings, more detailed mechanistic insight is lacking, mainly due to the lack of preclinical in vitro and in vivo models of ASCC. Of note, we have an established patient-derived organoid (PDO) research program for anorectal cancers in our center^46,47^ and have, hence, attempted to establish a PDO platform for ASCC to facilitate preclinical mechanistic studies to better understand the role of IFITM1 in ASCC (Goethe University of Frankfurt, Ethics committee protocol number 206/18). However, unlike PDO in rectal cancer patients, culture and growth of PDO from patients with ASCC was unsuccessful to date.

In conclusion, we found that chronic inflammatory pathways in tumor tissue, as measured by RNA sequencing (IFNγ; IFNα, TNFα signaling via NF-κB, IFITM1) and immunohistochemistry (IFITM1, PD-L1, CD8 + TIL), in line with elevated levels of regulatory T-cells and CXCL2 in peripheral blood, are associated with CRT resistance and poor prognosis in ASCC. One possible way to counteract the herein proposed resistance mechanism in ASCC could be the addition of immune checkpoint inhibitors to CRT. This hypothesis is currently tested in our ongoing, randomized phase-2 trial (RADIANCE) of standard CRT with or without addition of the immune checkpoint inhibitor durvalumab in locally advanced ASCC (NCT04230759)^48^.

## Methods

### Patients and treatment

Patients with histologically confirmed, localized ASCC, treated between 1998 and 2019 at 3 different sites (Frankfurt, Berlin, Tübingen) of the German Cancer Consortium (DKTK) were included. All patients received standard 5-FU/MMC-based CRT. Patient cohorts for molecular analysis were defined based on availability and quality of samples, including baseline formalin fixed paraffin embedded tissue for immunohistochemistry (FFPE, *n* = 130), baseline RNA sequencing of FFPE (*n* = 98), peripheral blood immune profiling (*n* = 47), and baseline serum cytokine measurement (*n* = 35). Patients and tumor characteristics of these cohorts are summarized in Supplementary Table 1. Due to the unavailability of sufficient tissue samples from the pretreatment biopsies, only a subset of 24 patients of the peripheral blood immune profiling cohort were available for tissue analysis. A flow chart depicting the composition and overlaps between these subsets is provided in Supplementary Fig. 1.

This study was performed in line with the principles of the Declaration of Helsinki. Approval for the collection of patient tissue and data within the DKTK-ROG was granted by the Ethics Committee of University of Frankfurt (Protocol Number 458/17). Taking of sequential blood samples during CRT was approved by the Ethics Committee of University of Frankfurt (Protocol Number 75/17). Informed consent was obtained from all individual participants included in the study.

### Immunohistochemistry

Immunohistochemical staining of pretherapeutic biopsies was performed by a horseradish-peroxidase technique using a fully automated DAKO Omnis staining system (Agilent, Santa Clara, CA, US) with standardized DAKO EnVision™ FLEX TRS Blocking reagent for antigen retrieval (DAKO, pH high, 97 °C) and polyconal IFITM1 (Sigma-Aldrich, Munich, Germany, dilution 1:250), monoclonal E-Cadherin (clone NCH-38, GA05961-2, Agilent DAKO, ready to use), Vimentin (clone V9, GA63061-2, Agilent DAKO, ready to use), CD8 (clone C8/144B, GA62361-2, Agilent DAKO; ready to use), PD-L1 (clone ZR-3, GE00621-2, Agilent DAKO, ready to use) and p16^INK4a^ antibodies (clone MX007, MAD000690QD12, Vitro-Master Diagnóstica, Sevilla, Spain, ready to use). Next, dextran polymer conjugated horseradish-peroxidase (EnVision FLEX HRP, DAKO) and 3,3’-diaminobenzidine (DAB) chromogen was used for visualization and DAKO hematoxylin solution (Agilent, Santa Clara, CA, US) for counterstaining. Negative control slides in the absence of primary antibodies were included for each staining.

For scoring of IFITM1, E-Cadherin and Vimentin, a weighted score was created by multiplication of the proportion of positive tumor cells (4: 75%–100%, 3: 50–75%, 2: 25–50%, 1: 0–25%) and the staining intensity (3: high, 2: medium, 1: low). The scores generated this way are between 1 and 12. The scoring of expression of CD8 + TIL was done semi-quantitatively as described before^14^. Categories for scoring the intratumoral component were: (1) no, or sporadic cells; (2) moderate number of cells; (3) abundant occurrence of cells; (4) highly abundant occurrence of cells. Patients were then divided into either having a high infiltration (score 3 or 4) or a low amount of infiltration (score 1 or 2). Analysis of p16^INK4a^ detection was done as described previously^45^. For PD-L1 tumor proportion score (TPS), immune cell score (IC) and combined positive score (CPS) were assessed^49^. Samples were independently evaluated by three investigators (DM, PKZ, FR) who were blinded to patient-specific clinical information. Images were acquired with the AxioScan Z.1 slide scanner and ZEN software (Carl Zeiss Microscopy, Jena, Germany).

### RNA sequencing

Baseline FFPE tumor samples were processed for total RNA isolation, library preparation and raw counts generation by GENEWIZ, LLC (South Plainfield, NJ, USA). Total RNA was isolated from FFPE samples using Qiagen RNeasy FFPE Kit (Qiagen) and depleted from ribosomal RNA (rRNA) to prepare sequencing libraries After quality control (TapeStation, Agilent Technologies, Palo Alto, CA, USA) samples were sequenced using a 2 × 150 paired end configuration (HiSeq 2 × 150 PE HO) and quality guarantee ≥80% of bases ≥Q3.

Overall analysis was done by a custom RNAseq analysis workflow, making use of public software packages. In this context, quality of raw reads was assessed prior and after trimming by FastQC (https://github.com/s-andrews/FastQC), while read quality trimming was performed by Trimmomatic version 0.39^50^. Trimmed and filtered reads were aligned on the Human Genome (Ensemble version hg38; homo_sapiens.104.mainChr.gtf) using the STAR mapper software (2.7.9a), using default parameters plus: --outFilterMismatchNoverLmax 0.1 --outFilterScoreMinOverLread 0.66 --outFilterMatchNminOverLread 0.66 --outFilterMatchNmin 20 --alignEndsProtrude 10 ConcordantPair --alignMatesGapMax 2000 --limitOutSAMoneReadBytes 10000000 --outMultimapperOrder Random --sjdbOverhang 100 --alignIntronMax 200000^51^. Counting of reads aligned to genes by STAR was performed by the featureCounts tool (included in the Subread package version 2.0.2)^52^. All reads that map at least partially within a given exon are aggregated to gene counts (homo_sapiens.104.mainChr.gtf). Multimapping reads were excluded. Following raw counting, gene expression values were normalized by the DESeq2 tool (1.30.0)^53^, and log transformed for further analysis. Differentially expressed genes were identified by DESeq2 as well (corrected *p* value of ≤0.05; Log2FC ≥ 0.58 and ≤−0.58 were considered to be differentially regulated).

Heatmaps were generated, using the normalized gene values and the complexheatmap R package, making use of *z*-score transformation per row. All groups of differential gene expression (DEG) lists were analyzed for enrichment of gene sets by the KOBAS tool (version 3.0) and the GSEA tool (version 4.3.2)^54^. For further exploration of the RNA sequencing data and DESeq2 analysis, patients were divided into two groups: poor responders who did either not have a clinical complete response (cCR) after treatment or developed locoregional or distant recurrence after initial cCR, and good responders who had a cCR and no recurrence during follow-up.

### Multiparametric flow cytometry of peripheral blood mononuclear cells

The prospective analysis of immune cells in the peripheral blood was done on a subset of 47 consecutive patients with available blood samples that were treated with standard CRT at Frankfurt. This prospective study was approved by the local ethics committee (Goethe University of Frankfurt, protocol Nr. 75/17) and written consent was obtained. Blood was taken before the initiation of treatment, on day 8 of CRT, on day 38 (last day of CRT), and on the first follow-up visit 6–8 weeks after completion of CRT.

Immune phenotyping was performed from peripheral blood mononuclear cells (PBMCs) following density gradient centrifugation (Biocoll, Biochrom, Berlin, Germany) by multi-color flow cytometry (CytoFLEX S, Beckman Coulter, Krefeld, Germany) using 3 panels of fluorescence-labeled antibodies (Supplementary Table 2). Briefly, 1 × 10^6^ cells were blocked with human FcR binding inhibitor (eBioscience, Frankfurt am Main, Germany), incubated with antibodies specific for lymphatic lineages (panel 1: CD45 [clone 2D1, 655873, dilution 1:10], CD3 [clone SK7, 332771, dilution 1:10], HLA-DR [clone L243, 655874, dilution 1:80]), T lymphocytes and activation markers (panel 2: CD3 [clone SK7, 332771, dilution 1:10], CD4 [clone SK3, 651849, dilution 1:80], CD8 [clone SK1, 641400, dilution 1:40), PD-1 [clone eBioJ105, 25-2799-42, dilution 1:40], Perforin [clone dG9, 556577, dilution 1:10]), or regulatory T cells (panel 3: CD3 [clone SK7, 332771, dilution 1:10], CD4 [clone SK3, 651849, dilution 1:80], CD8 [clone SK1, 641400, dilution 1:40], CD25 [clone 2A3, 740198, dilution 1:40], PD-1 [clone eBioJ105, 25-2799-42, dilution 1:40], FoxP3 [clone 236A/E7, 17-4777-42, dilution 1:40]) after fixation and permeabilization (Fix & Perm, Nordic MUbio, Susteren, Netherlands, and FoxP3 staining set, Invitrogen, Frankfurt am Main, Germany) for panels 2 and 3. Next, a total of 100,000 CD45+ cells were gated in case of panel 1, and of CD3+ cells in case of panels 2 and 3 (see Supplementary Fig. 2), and data were analyzed by using the CytExpert software (Beckman Coulter) with the ratio of positively stained cells defined as the percentage of cells within a defined leukocyte phenotype gate from total CD45+/CD3+ cells.

### Chemokines, cytokines and growth factor measurements in serum

Quantification of baseline chemokines, cytokines and growth factors protein levels in baseline blood serum (1:4 dilution) was conducted in a subset of 35 consecutive patients with available pretreatment serum samples. The Bio-Plex Pro human chemokine panel 40-plex Assay kit (Bio-Rad) was used according to the manufacturer’s instructions manual (#171AK99MR2) and analyzed using a Bio-PlexTM 100/200 reader (Bio-Rad).

### Statistical analysis

Differences between groups were assessed using Pearson’s Chi-squared test for categorical variables, and the nonparametric Wilcoxon rank sum test for continuous variables. Correlations between continuous variables were assessed using the Spearman’s rank correlation coefficient. Survival times were calculated from start of CRT to the date of respective events or last follow-up. Freedom from local failure (FFLF) was calculated using the date of diagnosis of incomplete response at restaging or locoregional recurrence after initial cCR as event. Freedom from distant metastasis (FFDM) was calculated using the date of diagnosis of distant metastases as event. Differences were calculated using the log-rank test or Cox regression analysis as necessary. The assumption of proportional hazard was verified by assessing the scaled Schoenfeld residuals. Cut-offs for IFITM1 and CXCL2 reads were calculated using the maximally selected rank statistics, which calculate the most optimal cut-off for a continuous variable^55^. All statistical analysis was performed with R (Version 4.2.2)^56^. A *p* value ≤ 0.05 was considered significant.

### Reporting summary

Further information on research design is available in the Nature Research Reporting Summary linked to this article.

### Supplementary information

Supplemental Material

Reporting Summary

## Data Availability

RNA Sequencing data are deposited under GEO accession GSE254331. Other data are available upon reasonable request.

## Code availability

Custom code that was used in the generation or analysis of the datasets is available upon reasonable request.
