# Supplementary material for: Inflammatory pathways confer resistance to chemoradiotherapy in anal squamous cell carcinoma
Source: NPJ Precis Oncol. 2024 Apr 23;8:93. doi: 10.1038/s41698-024-00585-y (PMC11039690; doi:10.1038/s41698-024-00585-y)
Supplement: Supplementary file 1 — Supplemental Material [file 41698_2024_585_MOESM1_ESM.pdf]

Supplementary Table 1: Clinical characteristics of RNA seq cohort, Immune blood profiling cohort and cytokine cohort

		Median (range) or n (%)		
		**RNA seq cohort**	**Immune blood profiling cohort**	**Cytokine cohort**
	n	98	47	35
Age, years		58 (35- 83)	59 (26- 83)	59 (40- 83)
Sex	male	45 (46)	24 (51)	19 (54)
	female	53 (54)	23 (49)	16 (46)
HIV-Status	positive	20 (20)	13 (28)	10 (29)
	negative	74 (76)	34 (72)	25 (71)
	unknown	4 (4)	0 (0)	0 (0)
T-Stage	T1	27 (28)	14 (30)	10 (28)
	T2	43 (44)	21 (45)	15 (43)
	T3	21 (21)	9 (19)	7 (20)
	T4	7 (7)	3 (6)	3 (9)
N-Stage	N0	72 (73)	27 (57)	18 (51)
	N+	26 (27)	20 (43)	17 (49)
Grading	G1	5 (5)	5 (11)	3 (9)
	G2	66 (67)	24 (51)	19 (54)
	G3	25 (26)	18 (38)	13 (37)
	unknown	2 (2)	0 (0)	0 (0)
Radiotherapy				
RT modality	3D	32 (33)	0 (0)	0 (0)
	IMRT	66 (67)	47 (100)	35 (100)
Total dose (Gy)		59.4 (45 – 65)	55.8 (53.2 - 59.4)	55.8 (53.2 - 59.4

Supplementary Table 2: Antibody panel for flow-cytometric analysis of peripheral blood mononuclear cells.

Antibody	Clone	Company	Fluorophore	Reference number
CD3	SK7	BD Biosciences	PerCP-Cy5,5	332771
CD4	SK3	BD Biosciences	V450	651849
CD8	SK1	BD Biosciences	APC-H7	641400
CD19	SJ25C1	BD Biosciences	PeCy7; PC7	341113
CD25	2A3	BD Biosciences	BV510	740198
CD45	2D1	BD Biosciences	V500	655873
PD-1 (CD279)	eBioJ105	eBioscience	PeCy7; PC7	25-2799-42
Perforin	dG9	BD Biosciences	FITC	556577
FoxP3	236A/E7	eBioscience	APC	17-4777-42
HLA-DR	L243	BD Biosciences	V450	655874

Supplementary Table 3: List of core enriched genes of the five enriched pathways associated with their regulation in the RNA seq cohort between cCR and PD patients.

**TNFa signalling via NF-KB**	**Upregulated in PD**	**Interferon Gamma Resp**	**Upregulated in PD**	**Interferon Alpha Response**	**Upregulated**	**Inflammator**	**Upregulated**	**Epithelial-mesenchym**	**Upregulated in PD**
SPSB1	no	IL6	no	PSME2	yes	ADRM1	yes	RHOB	yes
NINJ1	no	HLA-DQA1	no	RSAD2	no	TNFRSF1B	yes	CCN1	no
IL18	no	PNPT1	no	PROCR	yes	PLAUR	no	CCN2	no
BCL6	no	PARP14	no	RIPK2	no	ADORA2B	no	FBN1	no
IL6	no	OAS3	no	DDX60	no	BEST1	no	THY1	no
MAFF	no	HLA-A	no	IFI27	yes	SCN1B	no	COL1A2	no
CCL4	no	STAT1	no	ADAR	no	CCL24	no	COL8A2	no
ABCA1	no	PTPN6	yes	PSMB9	yes	GPR132	yes	LRRC15	no
HES1	no	CSF2RB	no	MX1	no	ADGRE1	yes	ADAM12	no
MYC	yes	GZMA	yes	SAMD9L	no	CXCL10	yes	TFPI2	no
CCL20	no	PSME2	yes	LAP3	yes	PDPN	no	CDH11	no
RHOB	yes	RSAD2	no	UBE2L6	yes	SPHK1	yes	GADD45B	yes
SERPINB2	no	RIPK2	no	IRF9	no	EMP3	no	FERMT2	no
CCN1	no	MX2	no	TRIM21	no	CD40	no	COMP	no
RIPK2	no	DDX60	no	USP18	no	IL18RAP	yes	FBLN2	no
GADD45B	yes	IFI27	yes	PSMB8	yes	PCDH7	yes	LOXL1	no
TNF	no	ADAR	no	C1S	no	HAS2	yes	CALD1	no
DENND5A	yes	PSMB9	yes	HLA-C	no	TNFAIP6	no	PLAUR	no
PLAUR	no	MX1	yes	CXCL10	yes	PROK2	no	TNFAIP3	no
PFKFB3	no	SAMD9L	no	TAP1	yes	RGS16	no	SAT1	no
TNFAIP3	no	C1R	no	TRAFD1	yes	EREG	no	SGCD	no
GFPT2	no	PFKP	yes	EPSTI1	yes	SCARF1	yes	COL3A1	no
SAT1	no	TNFAIP3	no	LGALS3BP	yes	FPR1	yes	ITGB5	no
KLF2	no	LAP3	yes	IFI44L	yes	OLR1	yes	IGFBP4	yes
MSC	yes	UBE2L6	yes	STAT2	yes	TLR2	yes	COL12A1	no
CLCF1	no	IRF9	no	CMTR1	yes	MEFV	yes	LAMC1	no
CEBPD	yes	TRIM21	no	GMPR	no	TNFSF9	yes	COL16A1	yes
MAP2K3	yes	USP18	no	DHX58	yes	HPN	yes	ITGB1	yes
TNIP1	yes	PSMB8	yes	IFITM3	yes	PVR	yes	CTHRC1	no
TNC	no	C1S	no	IFI35	yes	CSF3R	yes	COLGALT1	no
NR4A2	no	NOD1	no	IFI44	yes	TIMP1	yes	TGFB1	yes
PPP1R15A	no	CXCL10	yes	IFIH1	yes	CXCL6	yes	PPIB	yes
DRAM1	no	RBCK1	yes	OGFR	yes	CD14	yes	TNC	no
CXCL10	yes	TAP1	yes	HELZ2	yes	LIF	yes	FLNA	yes
TRIP10	yes	TRAFD1	yes	BST2	yes	AXL	yes	HTRA1	yes
SPHK1	yes	MVP	yes	IRF7	yes	MMP14	yes	PLOD1	yes
EFNA1	yes	EPSTI1	yes	IFIT3	yes	ITGA5	yes	THBS2	no
PANX1	yes	PSMB10	yes	LY6E	yes	IL1A	yes	PLOD3	yes
TAP1	yes	CD40	no	ISG15	yes	FFAR2	yes	EFEMP2	no
TNIP2	yes	HLA-B	yes	IFIT2	yes	BST2	yes	PDLIM4	yes
PLEK	yes	TNFAIP6	no	BATF2	yes	ICAM1	yes	BMP1	no
SLC2A6	yes	LGALS3BP	yes	IFITM1	yes	SERPINE1	yes	EMP3	yes
DUSP4	yes	ZNFX1	yes	CXCL11	yes	IRF7	yes	COL1A1	no
TNFAIP6	no	SECTM1	yes			LY6E	yes	LGALS1	yes

IER5	yes	IFI44L	yes			IL1B	yes	NID2	yes
NFKBIE	yes	STAT2	yes			INHBA	yes	COL5A2	no
OLR1	yes	RNF213	yes			F3	no	MFAP5	no
EHD1	yes	FPR1	yes			CXCL8	yes	PDGFRB	yes
DUSP5	yes	CMTR1	yes			CHST2	yes	P3H1	yes
SOCS3	yes	DHX58	yes			OSM	yes	COL6A3	no
CCND1	yes	SOCS3	yes			NLRP3	yes	GREM1	no
TLR2	yes	IFITM3	yes			AQP9	yes	COL4A1	yes
FJX1	yes	IFI35	yes			DCBLD2	yes	LAMA3	yes
SQSTM1	yes	XAF1	yes			CSF3	yes	COL5A1	yes
CEBPB	yes	IFI44	yes			IFITM1	yes	POSTN	no
TNFSF9	yes	IFIH1	yes			CXCL11	yes	PCOLCE	yes
NFKB2	yes	LATS2	yes					LOXL2	yes
IFIH1	yes	HLA-DRB1	yes					NNMT	no
CSF2	no	MT2A	yes					SPP1	yes
JAG1	yes	OGFR	yes					PVR	yes
LAMB3	yes	HELZ2	yes					TIMP1	yes
IRS2	yes	BST2	yes					MYL9	yes
PLAU	yes	IFIT1	yes					FAP	no
CXCL6	yes	ICAM1	yes					MMP1	yes
LIF	yes	IRF7	yes					TNFRSF12A	yes
GEM	yes	IFIT3	yes					CXCL6	yes
PTX3	no	LY6E	yes					LAMC2	yes
IL1A	yes	ISG15	yes					COL6A2	yes
ICAM1	yes	CFH	yes					COL4A2	yes
CXCL2	yes	IFIT2	yes					TGM2	yes
SERPINE1	yes	BATF2	yes					MMP3	yes
PMEPA1	yes	CXCL11	yes					GEM	yes
IL1B	yes							MMP14	yes
INHBA	yes							TGFBI	yes
F3	no							PTX3	no
CXCL1	yes							ITGA5	yes
G0S2	yes							SERPINH1	yes
AREG	yes							COL5A3	yes
BCL2A1	yes							SERPINE1	yes
IFIT2	yes							LAMA1	yes
CXCL11	yes							FSTL3	yes
CXCL3	yes							FN1	no
								PMEPA1	yes
								THBS1	yes
								INHBA	yes
								CXCL8	yes
								CXCL1	yes
								AREG	yes
								TPM2	yes
								NT5E	yes
								DKK1	yes
								VEGFC	yes

Supplementary Table 4: Percentage of CD3+ T-cells in FACS analysis of peripheral blood.

CD3+ T-cells	d1	d8	d38	d90
A001	44,36	53,04	4,21	22,02
A004	4,53	34,59	22,4	23,54
A003	11,52	46,76	12,89	15,72
A005	7,72	39,45	14,59	7,23
A006	28,90	31,56	7,81	12,23
A007	37,39	22,76	28,54	-
A008	11,54	21,12	7,87	20,7
A009	25,53	25,39	15,5	10,41
A011	45,71	48,07	20,33	29,21
A010	16,27	27,76	30,05	13,35
A012	29,94	33,7	9,78	4,53
A013	6,03	2,86	12,44	6,16
A014	30,80	20,02	20,59	28,66
A015	36,27	42,44	29,53	-
A016	37,10	30,53	11,51	18,43
A017	28,60	19,13	11,27	0,2
A019	37,49	30,37	16,28	-
A020	24,04	18,16	2,67	11,32
A018	50,23	40,37	9,54	27,94
A021	29,86	35,14	13,98	11,54
A022	24,37	18,72	9,92	17,51
A023	23,40	19,36	0,25	2,95
A024	46,46	4,81	0,46	18,69
A025	29,68	30,79	18,09	18,77
A026	20,23	12,84	14,13	21,66
A027	45,48	39,39	20,26	24,75
A028	37,00	42,5	25,15	10,53
A029	45,35	37,77	9,04	9,6
A031	24,71	34,59	10,21	-
A032	30,92	25,71	6,14	-
A030	3,87	19,48	18,54	27,23
A034	39,03	26,81	14,69	22,87
A033	20,32	17,77	-	11,57
A035	28,90	31,19	10,44	14,81
A036	35,79	41,31	12,23	33,19
A037	13,79	12,54	13,2	5,97
A039	22,79	21,15	10,72	23,75
A040	16,76	10,72	3,01	14,72
A043	22,85	-	11,22	-
A042	23,59	-	8,81	13,29
A044	34,34	-	7,71	18,01
A046	4,41	-	0,61	2,4
A045	14,79	-	5,45	-
A047	34,56	-	10,58	25,23

A048	47,80	-	17,05	38,1
A049	44,17	-	21,8	37,89
A050	30,62	-	7,12	10,63

Supplementary Table 5: Percentage of HLA_DR+ CD3+ T-cells in FACS analysis of peripheral blood.

CD3+HLA-DR+ T-cells	d1	d8	d38	d90
A001	14,05	14,38	37,94	56,24
A004	39,29	29,12	48,09	41,67
A003	15,26	10,14	24,78	72,03
A005	23,85	16,48	26,05	62,62
A006	14,29	9,49	17,51	29,24
A007	22,02	19,5	69,65	-
A008	28,18	22,12	36,83	67,13
A009	21,48	21,58	31,01	56,56
A011	24,24	19,86	47,28	55,45
A010	16,52	15,1	86,51	63,52
A012	33	23,83	53,3	81,15
A013	50,9	54,65	87,46	58,65
A014	5,99	9,95	16,81	53,45
A015	59,8	50,79	80,82	-
A016	8,48	7,81	21,31	42,56
A017	17,8	9,99	31,62	52,94
A019	8,97	9,14	10,44	-
A020	9,18	9,93	25,4	58,39
A018	6,18	5,14	11,1	41,56
A021	13,15	5,81	20,42	34,38
A022	10,41	12,67	37,84	38,54
A023	6,43	5,5	16,99	29,37
A024	13,76	11,24	95,2	59,24
A025	15,7	10,07	24,51	57,21
A026	26,28	21,58	47,95	64,11
A027	18,58	11,92	22,94	49,47
A028	10,51	8,89	16,08	26,5
A029	13,8	13,55	25,03	72,27
A031	10,5	13,42	27,61	-
A032	34,77	21,72	51,25	-
A030	40,44	34,02	67,2	44,53
A034	19,63	12,29	21,43	32,47
A033	10,71	8,65	-	31,05
A035	40,8	25,49	67,14	71,98
A036	48,48	48,9	73,12	79,06
A037	9,35	8,24	9,55	23,23
A039	57,72	57,07	43,92	99,96
A040	11,05	8,51	98,97	66,82
A043	99,94	-	31,73	-
A042	99,75	-	32,14	55,47

A044	28,22	-	24,3	67,6
A046	21,05	-	28,47	33,85
A045	17,7	-	30,57	-
A047	32,82	-	47,14	72,16
A048	17,59	-	19,94	72,49
A049	13,67	-	30,78	58,88
A050	6,16	-	18,32	22,09

Supplementary Table 6: Percentage of PD1+ CD4+ T-cells in FACS analysis of peripheral blood.

CD4+PD1+ T-cells	d1	d8	d38	d90
A001	14,94	21,28	17,31	30,17
A004	17,42	20,68	21,74	32,8
A003	10,45	20,87	23,24	48,19
A005	25,17	31,43	59,82	60,9
A006	13,97	15,68	26,08	26,95
A007	18,47	19,04	58,86	-
A008	17,83	22,87	41,87	56,49
A009	33,23	37,15	60,9	52,77
A011	24,2	22,2	31,04	49,86
A010	18,18	19,86	71,48	41,39
A012	27,49	24,34	34,53	41,45
A013	46,49	46,58	78,27	49,32
A014	13,81	19,73	24,85	29,87
A015	48,47	41,42	65,82	-
A016	36,64	15	35,98	20,93
A017	24,2	22,64	40,59	28,21
A019	15,39	15,78	24,03	-
A020	28,76	8,61	29,17	91,27
A018	10,9	12,14	19,92	84,29
A021	12,5	80,42	28,13	28,79
A022	89,93	83,12	44,06	47,21
A023	85,88	82,65	51,94	49,18
A024	78,06	42,53	41,2	47,3
A025	12,09	15,14	33,44	36,43
A026	32,67	38,41	63,53	61,14
A027	14,77	16,67	27,39	32,54
A028	13,1	12,53	23,95	24,94
A029	18,93	20,05	42,62	49,02
A031	13,07	12,1	31,02	-
A032	38,79	37,52	55,97	-
A030	25,23	31,29	45,94	42,3
A034	18,08	14,39	21,47	22,18
A033	14,32	13,89	-	48,09
A035	33,13	30,22	48,86	52,18
A036	20,9	20,84	42,32	32,75
A037	16	14,32	18,5	27,65
A039	20,28	31,34	24,03	55,2

A040	17,15	11,07	67,92	47,62
A043	16,31	-	32,79	-
A042	25,95	-	42,24	34,07
A044	22,67	-	31,27	47,21
A046	27,73	-	55,83	53,44
A045	12,13	-	26,62	-
A047	36,31	-	47	54,2
A048	8,87	-	17,23	26,71
A049	14,78	-	30,46	36,06
A050	11,53	-	17,85	25,34

Supplementary Table 7: Percentage of PD1+ CD8+ T-cells in FACS analysis of peripheral blood.

CD8+PD1+ T-cells	d1	d8	d38	d90
A001	43,2	48,47	20,99	48,78
A004	4,44	20,38	18,98	20,68
A003	28,73	47,16	34,44	40,04
A005	24,77	44,47	47,16	16,87
A006	18,8	17,48	24,62	35,83
A007	12,06	10,38	65,74	-
A008	20,73	28,17	36,26	46,82
A009	34,43	35,55	69,21	44,03
A011	46,95	37,55	36,28	54,78
A010	42,21	42,12	74,68	54,02
A012	19,86	16,97	9,83	27,78
A013	59,69	56,12	78,58	55,96
A014	13,17	15,48	30,55	34,85
A015	35,33	27,06	37,04	-
A016	42,03	21,9	33,27	19,96
A017	50,35	41,3	46,57	34,29
A019	18,66	9,06	21,99	-
A020	37,67	25,16	47,39	99,94
A018	12,82	21,98	24,63	99,98
A021	10,68	99,98	35,22	33,73
A022	99,88	99,86	36,5	38,38
A023	99,74	99,93	44,14	38,17
A024	99,95	94,71	34,52	32
A025	17,92	39,85	42,51	48,81
A026	25,71	39,32	69,86	54,45
A027	43,9	42,65	43,97	47,61
A028	28,75	30,33	32,05	28,08
A029	31,71	31,45	40,25	47,75
A031	26,52	33,41	38,99	-
A032	36,29	35,16	51,41	-
A030	33,37	39,5	36,42	24,05
A034	35,41	32,56	32,27	33,28
A033	40,1	39,95	-	66,79
A035	48,11	30,99	58,76	56,03

A036	35,98	31,82	41,08	8,73
A037	26,91	16,73	39,94	25,02
A039	37,27	36,41	31,7	24,97
A040	33,16	14,82	40,96	36,27
A043	20,25	-	47,97	-
A042	43,72	-	31,21	17,71
A044	37,46	-	37,17	57,75
A046	28,3	-	52,92	36,16
A045	31,18	-	42,18	
A047	25,77	-	36,87	29,08
A048	35,36	-	29,76	88,85
A049	43,59	-	41,73	87,74
A050	39,54	-	39,95	84,85

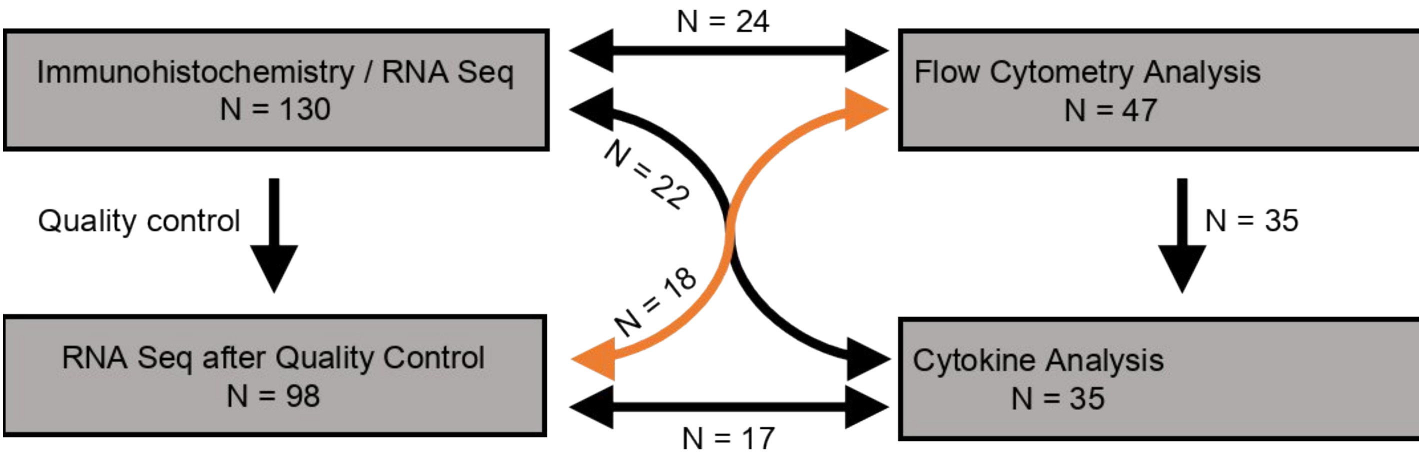

Supplemental Figure 1: Flowchart depicting the composition of the available samples in the different cohorts and the overlaps in between these cohorts. The numbers along the arrows represent the number of patients that overlap between the cohorts..

A

B

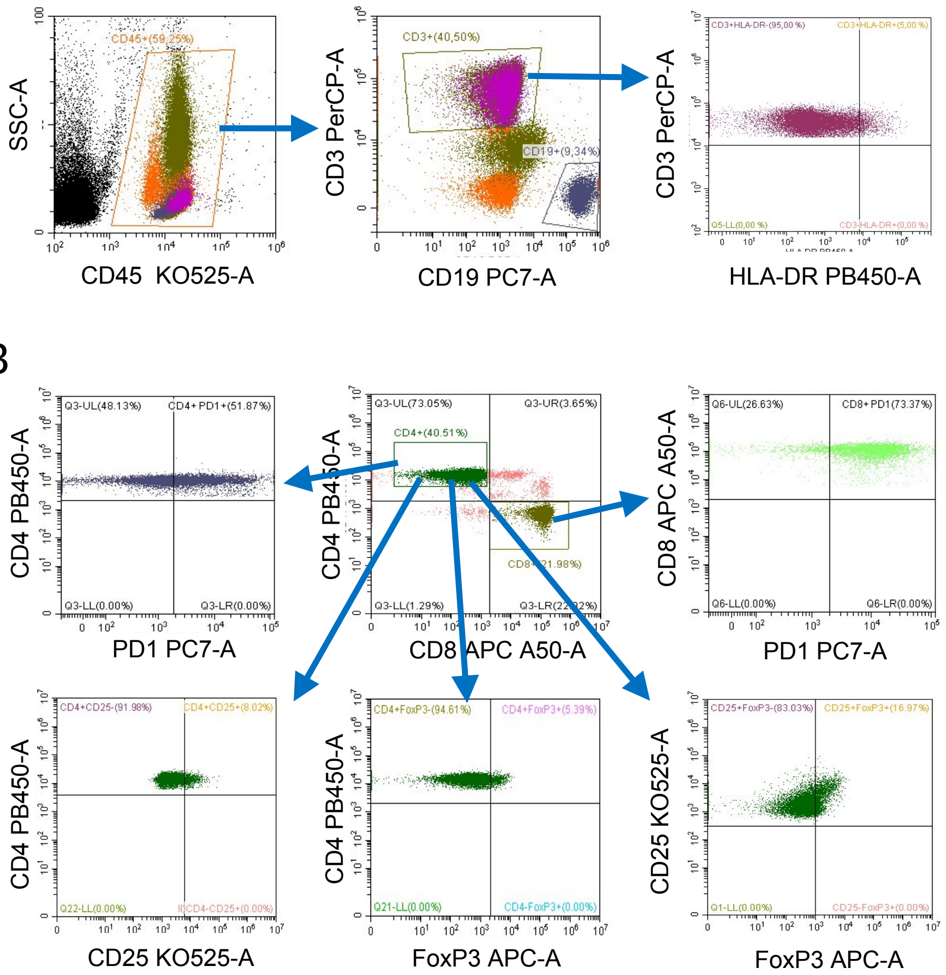

Supplemental Figure 2: Gating strategy for the cohort of n=47 patients with available blood samples during treatment. **A. Panel 1:** PBMCs (1 x 10^6) were stained with pan leukocyte marker CD45. Within this CD45+ population the T cells and the B cells cells were identified based on their surface expression markers CD3 and CD19. Activated T cells were determined with the expression of surface marker HLA-DR. **B. Panel 2:** PBMCs (1 x 10^6) were stained with CD45 and CD3 to identify T cells, followed by CD4 to identify T helper cells and CD8 to identify cytotoxic T cells and were further phenotyped by staining for PD-1/CD279 for checkpoint marker expression. CD4+ T cells were stained with CD25 and with anti-FoxP3 to identify T regulatory cells. Numbers represent the percentages from the parental population.

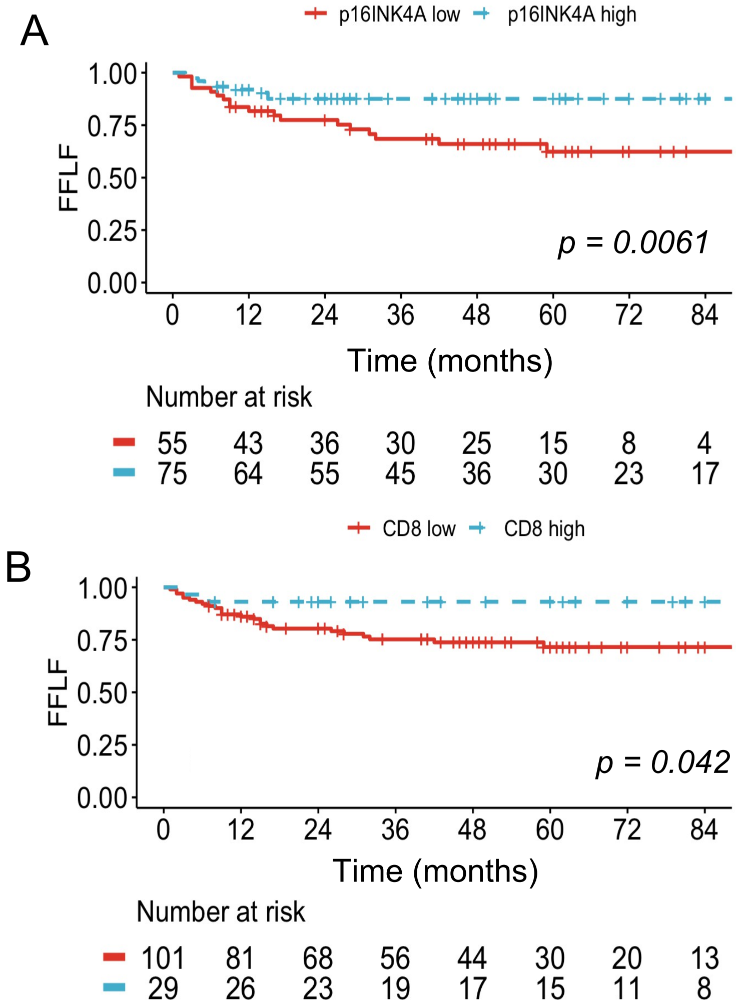

Supplemental Figure 3: A high expression of p16I^{NK4a} scored via immune histochemistry is associated with significantly increased freedom from locoregional failure (FFLF) (A). A high intratumoral infiltration with CD8+ tumor infiltrating lymphocytes (TIL) is associated with a higher FFLF.

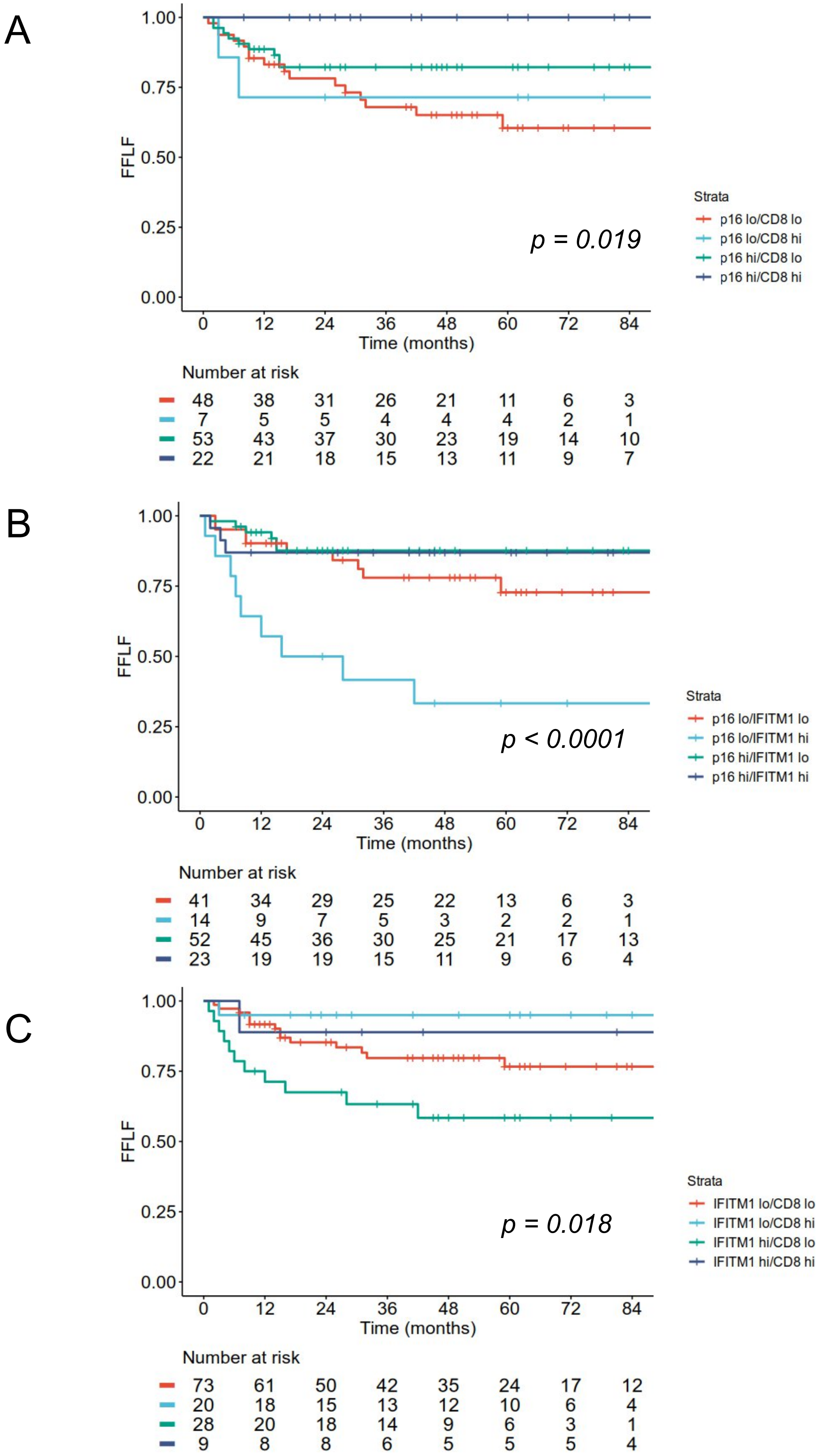

Supplemental Figure 4: Patients that had a high p16 score and a high infiltration with CD8 TIL had a significantly improved FFLF (A). Low p16 score and high IFITM1 expression were associated with a worse FFLF (B) whereas high IFITM1 expression and low infiltration with CD8 TIL was associated with worse FFLF (C).

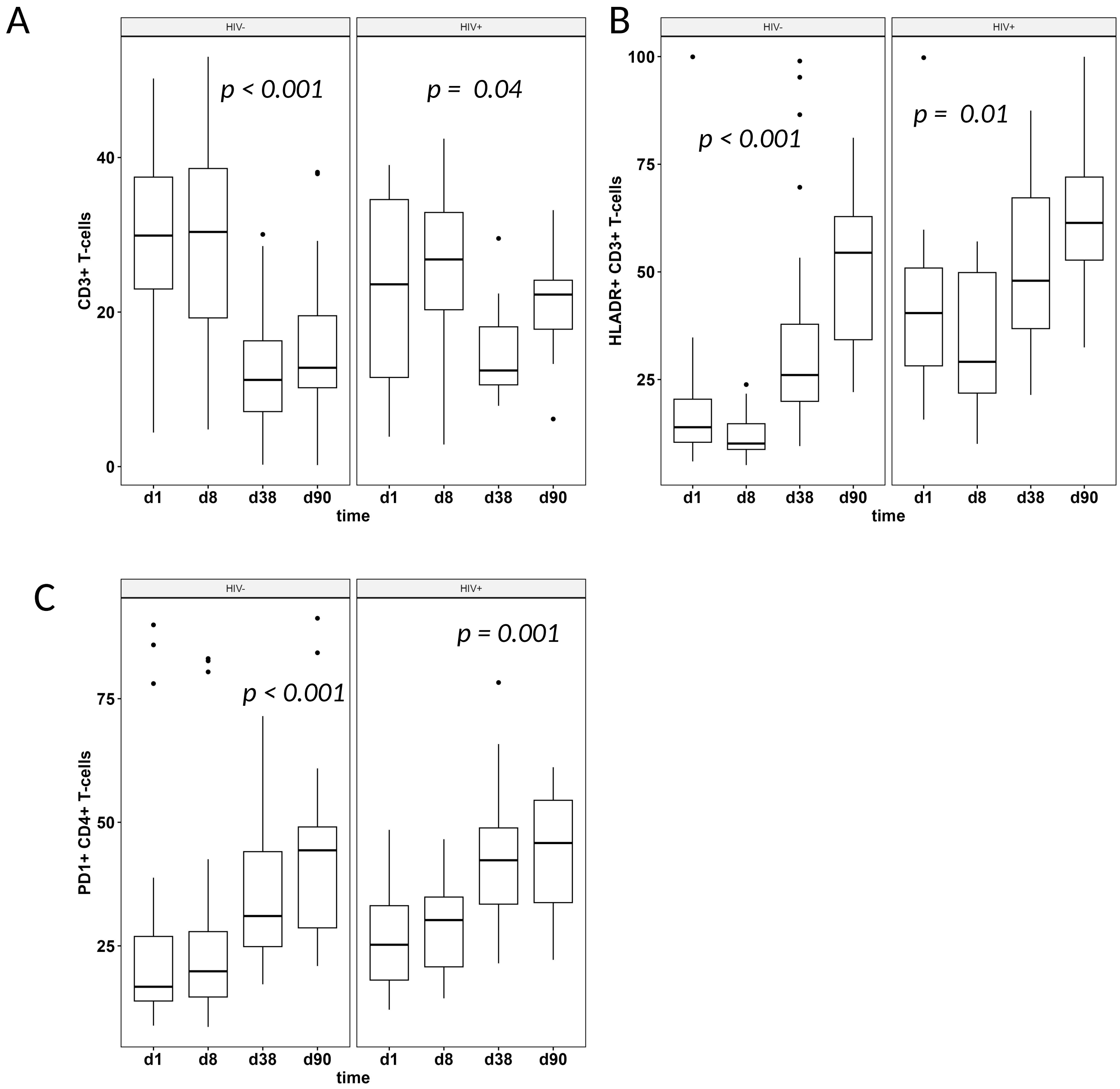

Supplemental Figure 5: Changes in CD3+ T-cells (A), CD3+HLADR+ T-cells (B) and CD4+PD1+ T-cells (C) were significantly independent of HIV status.

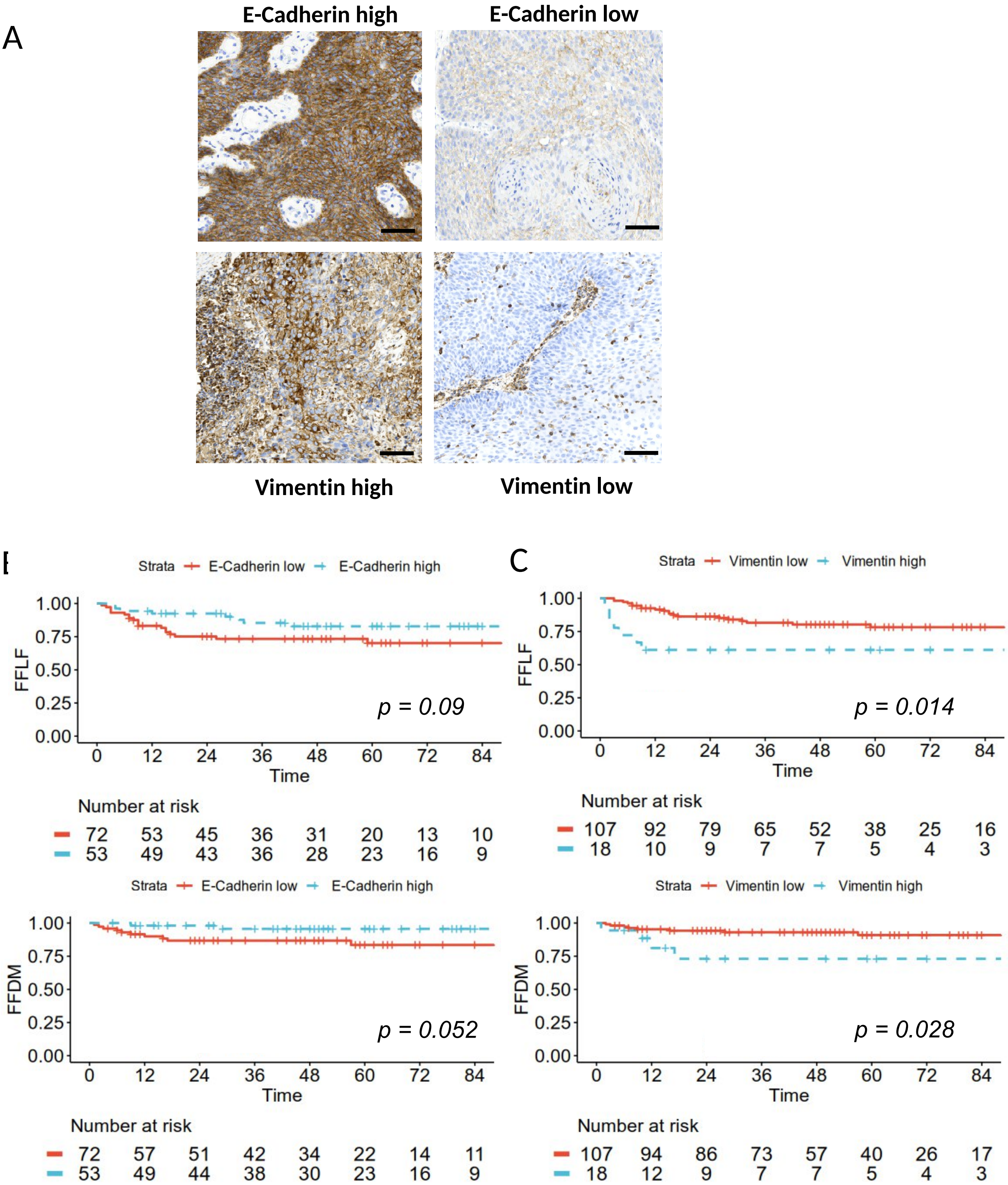

Supplemental Figure 6: Exemplary stainings of high and low expression of E-Cadherin and Vimentin (A). FFLF and FFDM for high and low expression of E-Cadherin (B) and Vimentin (C).
